# Controlled microstructure and mechanical properties of Al_2_O_3_-based nanocarbon composites fabricated by electrostatic assembly method

**DOI:** 10.1186/s11671-019-3061-4

**Published:** 2019-07-23

**Authors:** Wai Kian Tan, Norio Hakiri, Atsushi Yokoi, Go Kawamura, Atsunori Matsuda, Hiroyuki Muto

**Affiliations:** 1 Institute of Liberal Arts & Sciences, Toyohashi University of Technology, 1-1, Hibarigaoka, Tempaku-cho, Toyohashi, Aichi 441-8580 Japan; 2 Department of Electrical & Electronics Information Engineering, Toyohashi University of Technology, Toyohashi, Aichi 441-8580 Japan

**Keywords:** Nanocomposite, Electrostatic adsorption, Carbon microsphere, Alumina, mechanical property

## Abstract

This work reports on the microstructure-controlled formation of interconnected carbon-layered Al_2_O_3_ ceramics using carbon nanoparticles (CNP)-alumina (Al_2_O_3_) composite particles. The Al_2_O_3_ micro-particles used in this study were obtained by granulation of nano-sized Al_2_O_3_ nanoparticles with an average diameter of 150 nm. Then, CNP-Al_2_O_3_ composite was fabricated using an electrostatic assembly method using the granulated Al_2_O_3_ and CNP. The decoration of CNP on the surface of granulated Al_2_O_3_ was investigated as a function of primary particle size and coverage percentage using a fixed amount of CNP. Notably, an interconnected layer of carbon particles at the interface of Al_2_O_3_ that resemble the grain boundaries was obtained. The mechanical properties of the samples obtained with different particle size and CNP coverage on Al_2_O_3_ particles were also investigated which presented the possibility to control the mechanical properties through microstructural design of composite ceramic materials.

## Introduction

It is well known that alumina (Al_2_O_3_) possesses good properties such as high hardness, excellent wear resistance, and high chemical stability. On the other hand, the drawbacks of alumina are its poor fracture toughness, low strength at elevated temperature as well as poor thermal shock resistance [1]. This has prompted intense research in alumina-based nanocomposite development at micro- and nano-scales. Functional ceramic composites with well-dispersed nano-size particles in the ceramic matrix are reported to improve not only mechanical properties such as failure strength, fracture toughness, fatigue, and wear resistance but also the electrical, magnetic, thermal, and optical properties [2–7]. In order to improve and control the mechanical properties of ceramics, microstructural porosity [8, 9], incorporation of additive fillers [10], and heat-treatment profiles [11, 12] have been used and reported. This shows that by controlling the microstructure of Al_2_O_3_, the desired mechanical properties of Al_2_O_3_ ceramics could be obtained. However, most reported work merely used the simple method of Al_2_O_3_ powders mixing prior to sintering which is insufficient to obtain good control on the microstructure and design of Al_2_O_3_ ceramics resulting in poor controllability of its mechanical properties. In the formation of nanocomposite by a conventional mixing method, it remains a huge challenge to obtain a homogenous decoration of nano-sized additive particles onto a designated primary particle due to the additive particles agglomeration. The uneven distribution caused by the agglomeration would then lead to adverse effects on the microstructural design as well as the properties of a ceramic composite. Therefore, a novel method via bottom-up assembly using an electrostatic adsorption method was used in this study to demonstrate the feasibility to obtain a good microstructural control and design that consequently allow controlled desired properties to be introduced into Al_2_O_3_ ceramics such as optical, electrical, and mechanical properties. As one of the possible additives for Al_2_O_3_ ceramic composites, various shapes of nano-sized carbon materials such as fiber (carbon nanotube (CNT), nanofiber) and plate-like (graphene) as well as particle have been developed. This enables the application of carbon-based materials as an additive for materials fabrication which has been reported recently.

In the development of carbon-based alumina composite, Kumari et al. reported the enhancement of thermal conductivity of carbon nanotube (CNT)-alumina composite up from 60 to 318% compared to pure alumina by changing the weight percentage of CNT addition and sintering temperature [4]. Besides that, owing to the exceptional tribological properties of carbon-based composite materials for applications such as power generation, transportation, and manufacturing, many researchers have focused their interest into the development of carbon-based composites [13, 14]. Ceramics with carbon reinforced surfaces have been reported to exhibit improved wear resistance and a reduced friction coefficient. Despite controversial reports on mechanical strength enhancement using carbon nanofiber (CNF) on alumina and zirconia, most authors have reported improvement in the mechanical properties. A recent study of CNT on the creep property of alumina drew an opposing conclusion as it is reported that depending on the addition amount of CNT, the creep strength could be either strengthened or weakened due to an impediment of grain boundary sliding or promotion of grain boundary diffusion or sliding, respectively [15]. Meanwhile, Crepo et al. reported that graphene oxide-reinforced alumina composite exhibits better creep resistance than CNF-reinforced alumina [16]. Also, due to the excellent lubricating properties of graphite, carbon-based materials are a good candidate for solid lubricant application. During dry friction, carbon-based composites are reported to generate a lubricating film from the exfoliation of carbon and its incorporation with the ceramic debris over the affected contact area [13]. However, most of the reported work involves the usage of sole mixing by either ultrasonic mixing of suspensions or a conventional mechanical milling, and no work has been demonstrated on the controlled decoration of carbon materials on ceramic leading to the formation of microstructure-controlled carbon-based ceramics. Therefore, in this study, CNP-Al_2_O_3_ composites were fabricated using electrostatic adsorption assembly which offers more controllability in its composite assembly and design. The Al_2_O_3_ micro-particles used in this work were obtained using control granulation of nano-sized Al_2_O_3_ particles. Then, the granulated Al_2_O_3_ micro-particles obtained were used for the formation of carbon CNP-Al_2_O_3_ composite. The study was conducted systematically by varying the amount of carbon nanospheres from 0.3, 0.6, and 1.0 vol% (volume percent) and the average size of alumina particles used. The mechanical properties of carbon-based composite samples were then characterized and compared with a monolithic alumina sample using a three-point bending and indentation test. The inter-correlation between the microstructure obtained and mechanical properties is also discussed and elucidated.

## Methods

Alumina nanoparticles with an average diameter of 150 nm were purchased from Taimei Kagaku Kogyo Co. and used as the precursor to obtain granulated Al_2_O_3_ micro-particles. The granulation was carried out using spray drying of a mixture of Al_2_O_3_ micro-particles with acrylic binder. The Al_2_O_3_ micro-particles were then sieved to obtain three different average diameters 37, 62, and 98 μm which were then used as the primary particles. CNP with an average diameter of 260 nm was purchased from Tokai Carbon Co. and used as the additive nanoparticles. As CNP is hydrophobic and not dispersible in a water medium, it was first dispersed in SDC (sodium deoxycholate) solution and then subjected to a hydrophilization treatment for the subsequent coating. Forty milliliters of a 0.1 wt% SDC solution was added to 1 g of CNP and dispersed by ultrasonication for 30 min. Then, the solution was centrifuged and washed three times using ion-exchange water which was carried out by stirring the water with a mixer. After that, modification of the surface charge was carried out using polycation and polyanion. Polydiallyldimethyl ammoniumchloride (PDDA) (average molecular weight 100,000 to 200,000, Sigma-Aldrich) and polysodium styrenesulfonate (PSS) as polyanion (average molecular weight 70,000, Sigma-Aldrich) were used as the polycation and polyanion, respectively. After that, the SDC-coated CNP were then alternately immersed into PDDA, PSS, and PDDA in order to induce a stable positive surface charge. After the adsorption process, the remaining suspension was dried and then collected. In the first investigation, the Al_2_O_3_ particles with a diameter of 62 μm were used and the feasibility of CNP coverage control on Al_2_O_3_ particles was performed. The volume percentage of CNP added was 0.3, 0.6, and 1.0 vol%. In the investigation of the effect of Al_2_O_3_ size, a fixed 0.6 vol% of CNP addition was set while Al_2_O_3_ particles with different average diameters of 37, 62, and 98 μm were used for the composite formation. The CNP-Al_2_O_3_ composite particles were first uniaxially pressed using a die with a diameter of 12 mm. The pressure applied was 300 MPa and the holding time was 5 min. After that, the pellet obtained was inserted into a graphite die with h-BN powder for hot-press sintering (Diavac Inc. Ltd.) The hot-press sintering was carried out in a vacuum atmosphere (8 × 10^−3^ Pa) at 1350 °C (heating rate of 10 °C/min) for 2 h with a pressure of 30 MPa. The morphologies of the CNP-Al_2_O_3_ composites and the sintered microstructure obtained were observed using an S-4800 Field Emission Scanning Electron Microscope (FE-SEM, Hitachi S-4800). The zeta potential was measured using an Otsuka Electronics Co. Ltd., ELSZ-1 and Micro Tech Nission, ZEECOM Co. Ltd. As for the mechanical properties determination, the elastic modulus of the sample obtained was measured using a 3-point bending test. The sample was first cut into a strip-shaped test sheet and the dimension was fine-tuned using a surface grinder. The dimension of the test specimen prepared was 3 × 4 × 40 mm. After that, polishing was carried out using 0.5-mm alumina and diamond paste with a grade of 30 and 9 μm, respectively. The 3-point bending test was measured using an Instron type compact tester. First, the stress (*σ*) was calculated using Eq. 1 where, *l*, *b*, and *h* are the span distance and dimensions of each test piece, while *P* represents the load. Next, the relationship between stress and strain was plotted, and the elastic modulus was calculated from the slope of least squares. The crosshead speed was tested at 0.02 mm/min and the span at 30 mm.

1 $$ \sigma =\frac{3 lP}{2 bh} $$

The hardness properties of the composite sample were further evaluated using indentation. The Rockwell indenter used consisted of a diamond (*E*_i_ = 1050 GPa, *υ* = 0.20) with a nominal radius of curvature, *R* = 200 embedded in a conical tip with an apex angle of 120°. The indenter was set in an Instron type tester (Sanwa Instruments) and was driven in at a crosshead speed of 0.05 mm/s to a fixed depth (20 μm). The load obtained during indentation was measured with a load cell (TCLZ-100KA, Tokyo Gakko), and the indent depth was measured with a non-contact electrostatic displacement meter (VE-222, Ono Sokki).

## Results and Discussion

**Fig. 1:**
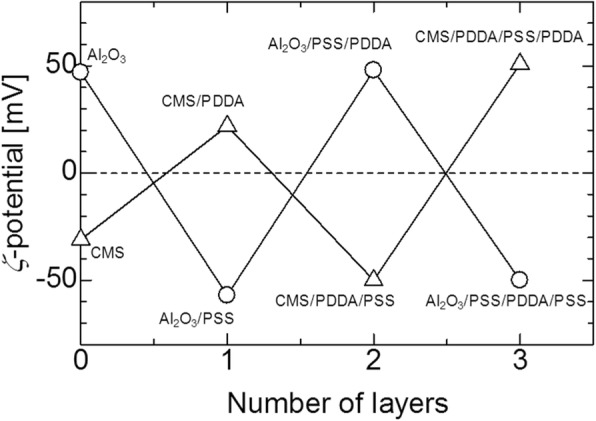
Zeta potential of surface charge-controlled Al_2_O_3_ and carbon nanoparticles

**Fig. 2:**
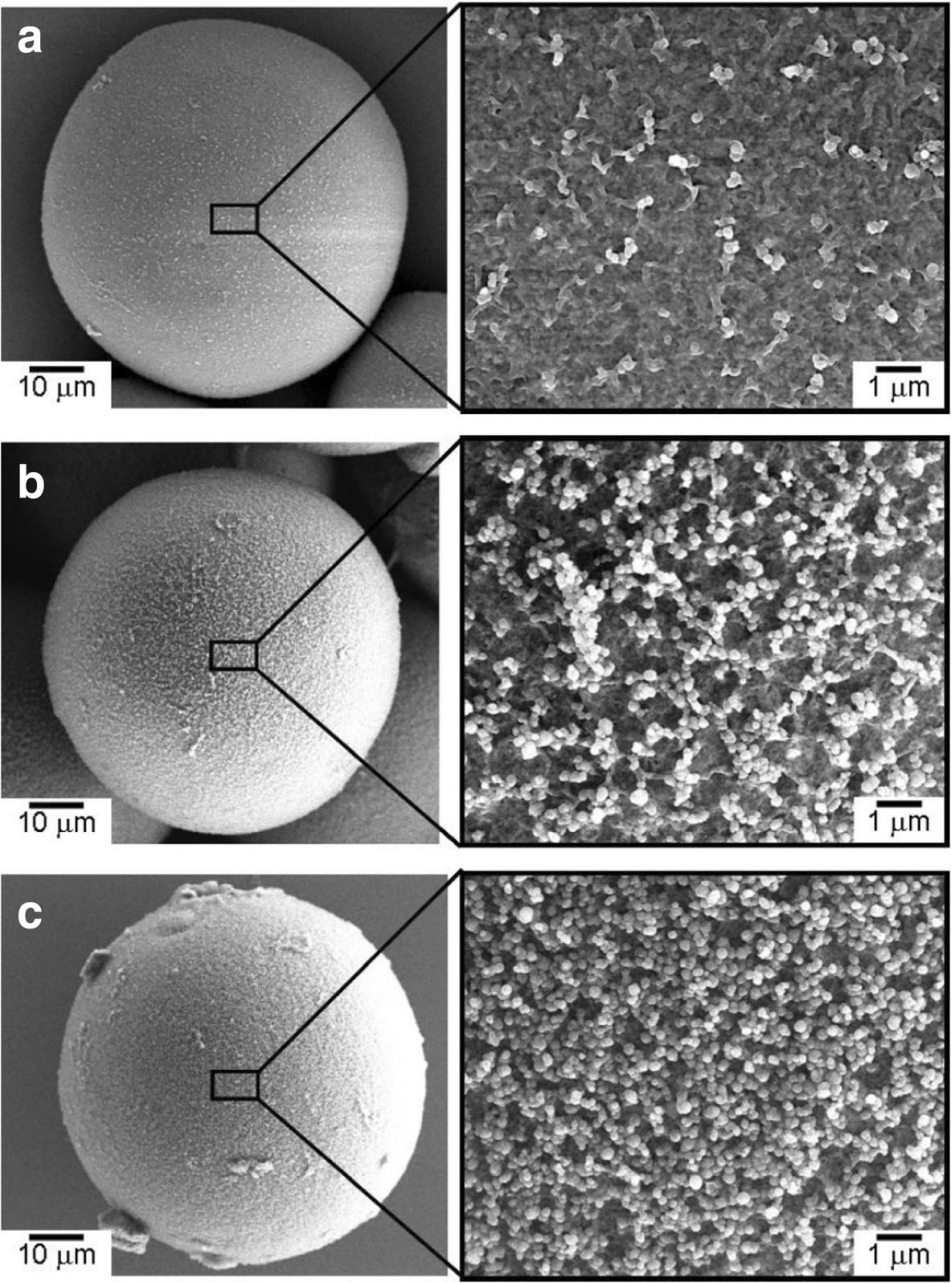
SEM images of the **a** 0.3 vol%, **b** 0.6 vol%, and **c** 1.0 vol% CNP coated on Al_2_O_3_ granulation particle with average diameter of 62 μm

**Fig. 3:**
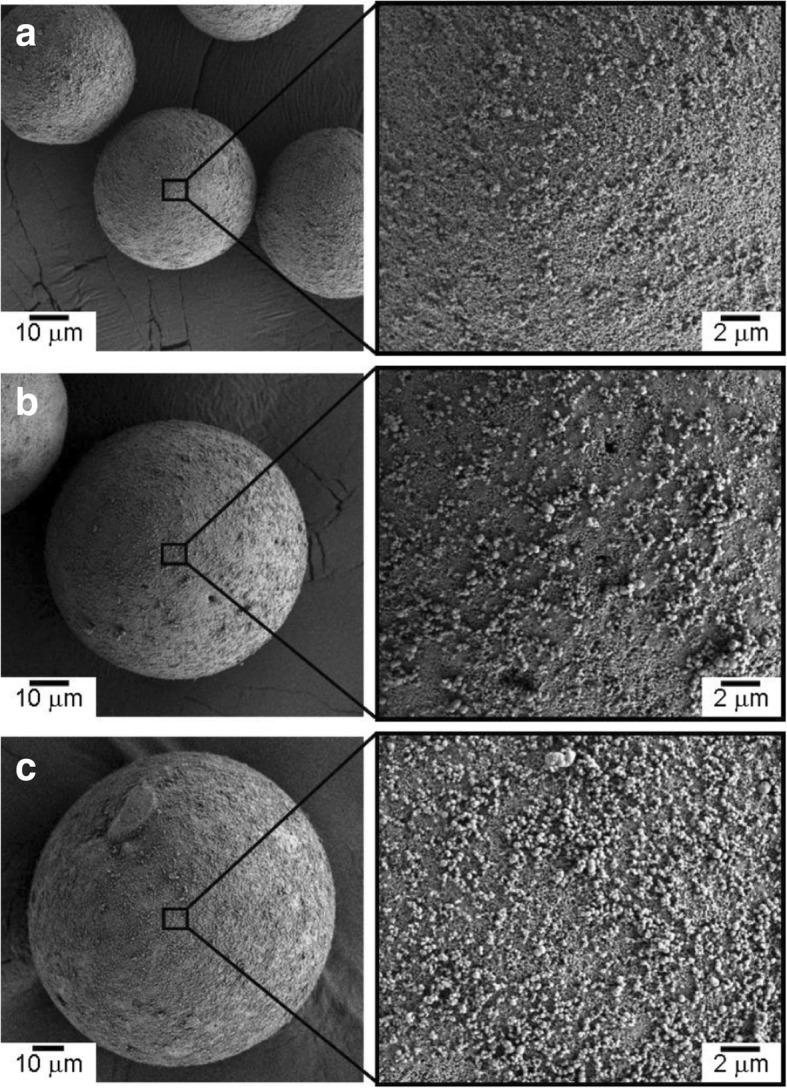
SEM images of the 0.6 vol% CNP coated on Al_2_O_3_ granulation particles with average diameter of **a** 37, **b** 62, and **c** 98 μm

**Fig. 4:**
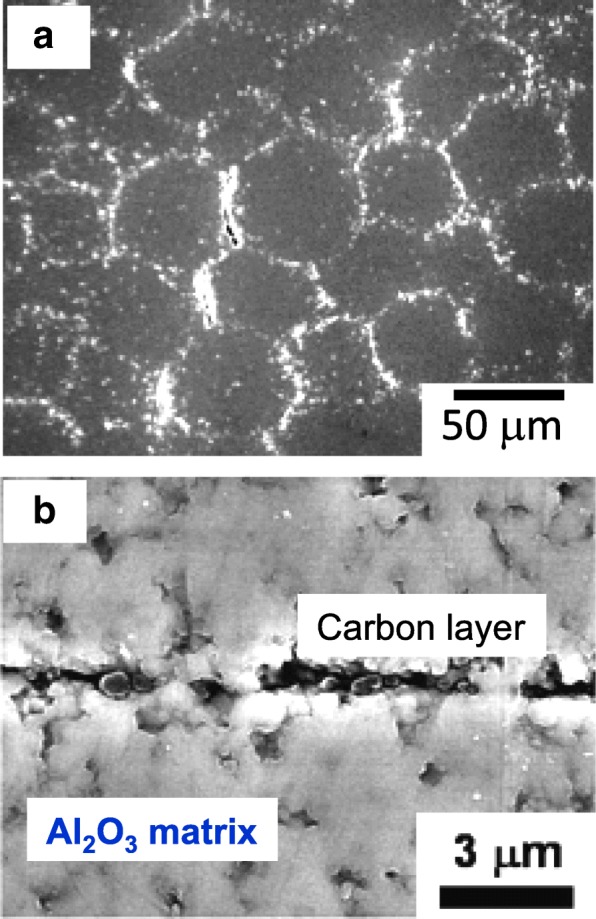
**a** Microstructure of 0.6 vol% CNP-Al_2_O_3_ composite using Al_2_O_3_ with the average diameter of 62 μm. **b** Grain boundary of CNP-Al_2_O_3_ composite. Carbon layer could be observed at the interface between the Al_2_O_3_ matrix

**Fig. 5:**
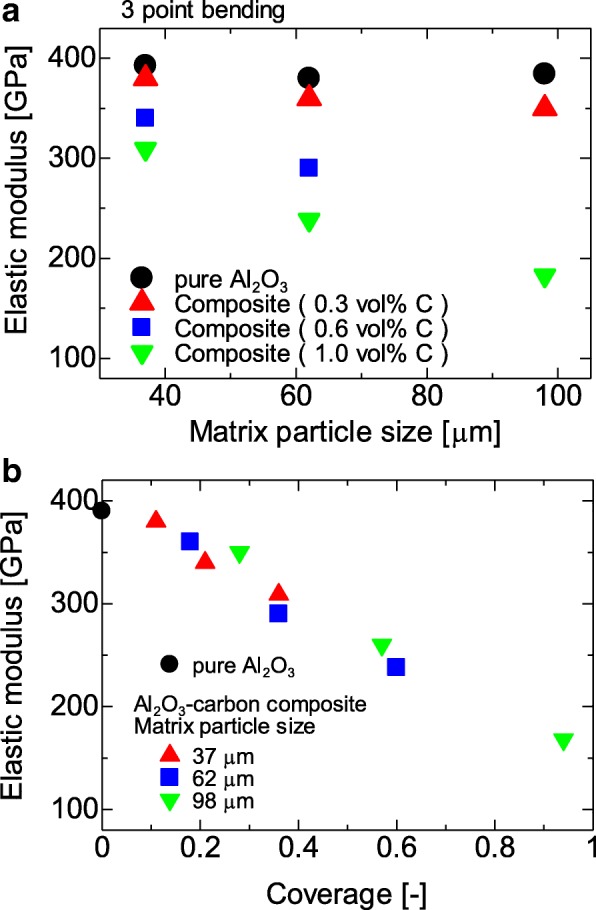
Elastic moduli of CNP-Al_2_O_3_ composites as a function of **a** matrix particle size and **b** CNP coverage on Al_2_O_3_ particles

**Fig. 6:**
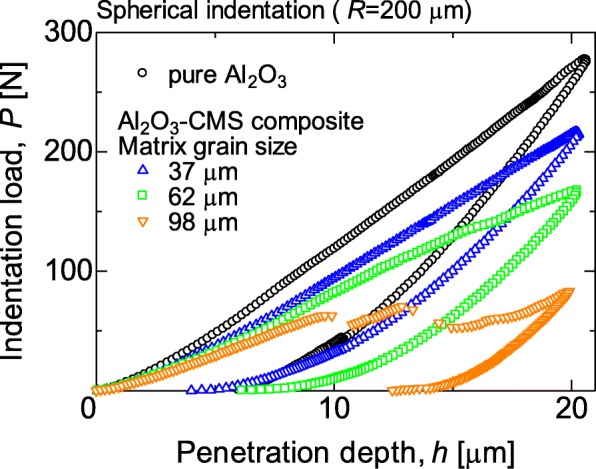
Hysteresis curves of indentation load and penetration depth of 1.0 vol% CNP-Al_2_O_3_ composites

Figure 1 shows the surface charge zeta potential of Al_2_O_3_ particles and CNP after alternating coatings of PDDS and PSS, accordingly. It could be observed that the alumina and CNP exhibited a zeta potential of + 55 and − 55 mV, respectively, after three layers of coating. The achieved zeta potential after three layers of coating on both CNP and Al_2_O_3_ micro-particles was stable. The surface morphologies of the CNP-Al_2_O_3_ composites with different 0.3, 0.6, and 1.0 vol% of CNP addition are shown in the SEM images of Fig. 2. From the higher magnification SEM images, it can be clearly observed that the amount of CNP that are adsorbed onto the surface of Al_2_O_3_ particle increased with a higher volume percent of CNP addition. It is important to note the CNP are distributed homogenously throughout the Al_2_O_3_ surface without sign of agglomeration which portrays the advantage of the EA method to obtain an even and uniform distribution. The particle size of the CNP observed is approximately 260 nm. By fixing the addition of CNP at 0.6 vol% and varying the size of the Al_2_O_3_ micro-particles from 37, 62, and 98 μm, the distributions of CNP on the surface of Al_2_O_3_ particle are shown in the SEM images in Fig. 3. From the observation of the SEM images, it could be seen that as the diameter of the particle size increased, the amount of CNP adsorbed on the surface was observed to increase accordingly. As larger Al_2_O_3_ particles possess lower overall surface area compared to the smaller Al_2_O_3_ particles, the collective surface area available for the adsorption of CNP is also lower compared to smaller Al_2_O_3_ particles. Therefore, with a constant of 0.6 vol% addition of CNP, a higher amount of CNP was adsorbed onto the lower overall surface area of larger Al_2_O_3_ particles. As a result, the amount of CNP adsorbed onto the Al_2_O_3_ surface is observed to increase with the diameter size of Al_2_O_3_ particles that led to a higher density of CNP adsorption onto the surface of 98 μm Al_2_O_3_ particles. On the other hand, as the particle size reduced, the available overall surface area accessible for CNP adsorption on Al_2_O_3_ increased and therefore, a sparse distribution of CNP is observed due to the insufficient amount of CNP in the suspension (at a fixed 0.6 vol%). The sintered microstructure obtained using the CNP-Al_2_O_3_ composite and high magnification at the interface as shown in Fig. 4. From the SEM image in Fig. 4a, it can be seen that the microstructure obtained reflects the shape of the obtained CNP-Al_2_O_3_ composite. It is noteworthy that the grain boundaries are connected forming a network along the grain boundaries. From the observation of the CNP network that is formed along the grain boundaries, the homogeneity of CNP distribution on the surface of Al_2_O_3_ particles can be determined. This result shows that it is feasible to obtain a microstructure-controlled composite material by designing the composite precursor. From the higher magnification SEM image in Fig. 4b, the presence of a carbon layer in between the interface of the Al_2_O_3_ grain boundary can be observed. This shows that the sintering of the CNP in between the Al_2_O_3_ particles during hot-press sintering led to the formation of an even coating of a carbon layer along the grain boundaries. It is also important to note that the Al_2_O_3_ matrix obtained is dense and well sintered with no observation of pores as shown in Fig. 4b. This is due to the formation of densely packed granulated Al_2_O_3_ nanoparticles (150 nm) which allows good sinter-ability that demonstrated the novel technique of this work. The elastic moduli of the CNP-Al_2_O_3_ composite obtained using a 3-point bending test plotted as a function of Al_2_O_3_ particle size and surface coverage percentage are shown in Fig. 5. From Fig. 5a, the exhibited elastic modulus of the sample fabricated using Al_2_O_3_ particles only is approximately 390 GPa which is consistent with the results reported on polycrystalline Al_2_O_3_ which is between 300 and 400 GPa [6, 12]. The achievement of this elastic modulus value corroborates with the SEM observation where a good microstructure and compaction was achieved using granulated Al_2_O_3_ nanoparticles. In the study of Ashizuka et al. on the effect of porosity on the mechanical properties of alumina ceramics, the elastic modulus of the ceramic without porosity (0%) is slightly lower at approximately 380 GPa [17]. As for the elastic moduli of the CNP-Al_2_O_3_ composites, it can be seen that the property could be controlled as it decreased linearly with either higher volume percent addition of CNP or increment in the Al_2_O_3_ particle size. A similar trend was also observed in the work of Shin et al., where the elastic moduli of their reduced graphene oxide and single-wall CNT-alumina composites were reduced by increasing the additive content [6]. As both factors (amount of CNP and particle size of Al_2_O_3_ ) highly influence the specific surface area and led to greater adsorption of CNP on the Al_2_O_3_ particle surface, this would inhibit the sintering of Al_2_O_3_ and a possible slipping effect of the carbon layer resulted in lower elastic modules [6]. This finding is consistent with those reported by Gopalan et al. where the CNT used in their composite retarded grain growth but had no effect on the grain boundary sliding resulting in the occurrence of superplasticity [15]. This finding indicates the possibility to alter and control the elastic modulus of an Al_2_O_3_ ceramic by controlling the microstructural formation via the design of the precursor composite used in the formation of CNP-Al_2_O_3_. In Fig. 5b, the plot of the obtained elastic moduli as a function of the CNP coverage ratio on Al_2_O_3_ is shown. A linear correlation between the CNP coverage ratio and elastic modulus strength is observed which further corroborate with the abovementioned results. Therefore, from these results, it is demonstrated that the mechanical properties of a CNP-Al_2_O_3_ composite ceramic can be controlled via the CNP coverage ratio by either altering the amount of CNP addition or the particle size of primary Al_2_O_3_. In the determination of the micro-hardness of the CNP-Al_2_O_3_ composite samples, a comparison between pure Al_2_O_3_ and CNP-Al_2_O_3_ samples fabricated with 1.0 vol% of CNP addition with different Al_2_O_3_ particle sizes of 37, 62, and 98 μm was undertaken. The indentation results obtained are shown in Fig. 6. The results obtained show that the pure alumina sample exhibited the highest hardness value while the hardness of CNP-Al_2_O_3_ composite samples reduced with larger Al_2_O_3_ particle size. This is due to the lower overall surface area of Al_2_O_3_ when the particle size increase leading to a higher amount of CNP adsorbed on the surface. Subsequently, the higher amount of CNP on the Al_2_O_3_ interface led to reduced hardness due to either the inhibition of an effective sintering between the Al_2_O_3_ interface or the slip of the continuous connected carbon layer along the grain boundaries of Al_2_O_3._ Therefore, it is crucial to have a controlled distribution of CNP on the surface of Al_2_O_3_ in order to induce the formation of a desired microstructure leading to the desired mechanical properties of CNP-Al_2_O_3_ composite.

From the indentation load (*P*)-depth (*h*) curve (*P*-*h* curve) during the loading and unloading cycle, microstructural change and mechanism of surface deformation could be obtained [18]. The simple quadratic equation involving indentation load (*P*) and penetration depth (*h*) shown in Eq. 2 can be used for the analysis of loading process [18–20].

2 $$ P\propto {h}^2 $$

The loading of monolithic Al_2_O_3_ demonstrated a correlation with the *P*-*h* curve similar to the quadratic Eq. 2 while CNP-Al_2_O_3_ composite fabricated using Al_2_O_3_ particle with the size of 37 and 62 μm demonstrated a linear with deviated curve from the monolithic Al_2_O_3_ loading curve, respectively. This indicates the presence of CNP within the microstructure (at the grain boundary interface) which resulted in local deformation along the grain boundaries. As for CNP-Al_2_O_3_ composite fabricated using Al_2_O_3_ with the particle size 98 μm, the high density of CNP at the grain boundaries resulted in discontinuity of *P*-*h* hysteresis curve and demonstrated the lowest hardness due to occurrence of grain boundary slip or surface microfracture.

## Conclusions

In this work, a feasible controlled formation of CNP-Al_2_O_3_ composite by an electrostatic adsorption method is demonstrated. The Al_2_O_3_ micro-particles used were obtained by granulation of nano-sized (150 nm) Al_2_O_3_ particles which enabled better compaction and sinter-ability. In the formation of composite ceramics, parameters involving the amount of CNP (0.3, 0.6, 1.0 vol%) and primary granulated Al_2_O_3_ micro-particle sizes (37, 62, 92 μm) were investigated. It is demonstrated that by controlling the amount of CNP additives and Al_2_O_3_ micro-particle size, different surface coverage could be obtained leading to controlled microstructure formation with different mechanical properties. Using the homogenous CNP-Al_2_O_3_ composite, a continuous interconnected carbon layer was obtained along the grain boundaries of Al_2_O_3_. A dense and compact Al_2_O_3_ matrix was also observed due to the good sintering of Al_2_O_3_ nanoparticles. From the results of a 3-point bending and indentation test, the control of mechanical properties was demonstrated by adjusting the coverage of CNP on Al_2_O_3._ The change in elastic modulus was either due to the inhibition of effective sintering or the slipping of the carbon layer generated at the Al_2_O_3_ interface. From this study, we have demonstrated the feasibility of ceramics microstructural design with an interconnected interface using CNP-Al_2_O_3_ composite. This method of microstructural design will open up greater possibilities and potential for materials design through bottom-up assembly to induce the desired properties for a wide range of applications.

## Data Availability

All data generated or analyzed during this study are included in this published article (and its supplementary information files).

## Abbreviations

CNP: Carbon nanoparticles
PDDA: Polydiallyldimethyl ammoniumchloride
PSS: Polysodium styrenesulfonate
SDC: Sodium deoxycholate
SEM: Scanning electron microscope
