# Correction to: Controlled Microstructure and Mechanical Properties of Al2O3-Based Nanocarbon Composites Fabricated by Electrostatic Assembly Method

**DOI:** 10.1186/s11671-019-3122-8

**Published:** 2019-08-19

**Authors:** Wai Kian Tan, Norio Hakiri, Atsushi Yokoi, Go Kawamura, Atsunori Matsuda, Hiroyuki Muto

**Affiliations:** 1 Institute of Liberal Arts & Sciences, Toyohashi University of Technology, 1-1, Hibarigaoka, Tempaku-cho, Toyohashi, Aichi 441-8580 Japan; 2 Department of Electrical & Electronics Information Engineering, Toyohashi University of Technology, Toyohashi, Aichi 441-8580 Japan

**Correction to: Nanoscale Res Lett**

**http://dx.doi.org/10.1186/s11671-019-3061-4**

Please note that in the original article [1] the name of the second author, Norio Hakiri, was erroneously ordered; the name was ordered with the given and family names the wrong way around, as ‘Hakiri Norio’.

The name has subsequently been corrected in the original article.

## References

[1] Tan (2019). Controlled microstructure and mechanical properties of Al2O3-based nanocarbon composites fabricated by electrostatic assembly method. Nanoscale Res Lett.

